# In-vitro experimental evaluation of skin-to-surface recovery of four bacterial species by antibacterial and non-antibacterial medical examination gloves

**DOI:** 10.1186/2047-2994-2-27

**Published:** 2013-10-11

**Authors:** Johannes Leitgeb, Rupert Schuster, Aik-Hwee Eng, Bit-New Yee, Yee-Peng Teh, Verena Dosch, Ojan Assadian

**Affiliations:** 1 Department for Trauma Surgery, Medical University of Vienna, Waehringer Guertel 18-20, A-1090 Vienna, Austria; 2 Ansell Healthcare Products LLC, S&T Innovation Centre, 40000 Shah Alam Selangor, Malaysia; 3 Department for Hospital Hygiene, Medical University of Vienna, Waehringer Guertel 18-20, A-1090 Vienna, Austria

**Keywords:** Examination glove, Surface contamination, Antimicrobial glove, Polyhexanide, PHMB, Cross-contamination

## Abstract

**Background:**

The number of bacteria recovered from a stainless steel coupon after touching a pigskin substrate with an examination glove coated on its outside with polyhexanide (PHMB), as compared to the number of bacteria recovered in the same manner with non-coated control gloves was evaluated.

**Methods:**

Suspensions containing 1 × 10^9^ colony-forming units of 4 clinically relevant bacterial species (*Enterococcus faecium* ATCC #51559; *Escherichia coli* ATCC #25922; *Klebsiella pneumoniae* ATCC #4352; and *Staphylococcus aureus* ATCC #33591) were used to contaminate Gamma-irradiated pigskin substrates. Bacterial recoveries from the pigskin substrate, stainless steel coupons, and each glove swatch were performed. A difference in the bacterial recovery from the stainless steel coupons after touching with coated and uncoated control gloves was measured.

**Results:**

For *E. faecium*, the coated glove showed a reduction of 4.63 log_10_ cfu recovery, when compared to control gloves. For *E. coli*, the coated glove showed 5.48 log_10_ cfu, for *K. pneumoniae* 5.03 log_10_ cfu, and for *S. aureus* 5.72 log_10_ cfu recovery, when compared to the non-coated control glove.

**Conclusion:**

An in-vitro experiment designed to mimic cross-contamination of clinically relevant bacteria in a simulated healthcare setting following glove contact with a contaminated biological surface and cross-transfer to a stainless steel surface has demonstrated that an examination glove coated on its outside surface with PHMB was able to reduce bacterial recovery from a contaminated surface by > 4 log_10_ cfu, compared to a control non-coated examination glove. These elaborated results may encourage further clinical investigation on the clinical impact of an antibacterial examination glove.

## Introduction

Medical examination gloves are used by healthcare workers for two main reasons: to reduce the risk of contamination of the healthcare workers’ hands during patient care, and to reduce the risk of direct microbial dissemination to the environment and hence, further indirect transmission of pathogenic bacteria to other objects or individuals/ patients. While the first objective depends on the glove’s integrity, prevention or reduction of bacterial dissemination from one source to other surfaces will be achieved only if such gloves are used correctly.

A single-use medical examination glove is used correctly if it is donned on hands before caring for a patient or before manipulation of innate or viable surfaces, which are anticipated to harbour pathogenic microorganisms in high numbers. Such situations typically occur when mucous membranes or wounds are touched, or during all patient-care activities involving exposure to blood or body fluids that may be contaminated with microorganisms. Although it is evident that a single-use examination glove must be used only on one patient, in clinical practice it may occur that healthcare workers don a new pair of examination gloves, care for one single patient, however, while doing so, eventually may touch a surface close to the patient, and continue to care for the same patient. At the end of patient care, gloves are taken off, and hands are disinfected. When healthcare workers care for the next patient adjacent to the first patient, they will use a new pair of examination gloves, start to care for the second patient, and eventually may unintentionally touch the previously contaminated surface situated between the two patients. Hence, a cross-transmission from one patient to the other via the contaminated surface and the contaminated examination gloves may result.

Therefore, immediately after touching a contaminated surface, and before any other surface in the surrounding is touched, the contaminated medical glove must be taken off from hands, and hand disinfection must be performed.

Strict adherence to the above procedure requires knowledge on bacterial transmission, training, great attention, and a high level of concentration during clinical work. It was highlighted by the WHO [1] that the broad scope of these recommendations for glove use together with the significant increase of usage frequency potentially lead to inevitable, undesirable consequences, such as the misuse and the overuse of gloves, resulting in bacterial dissemination to the surrounding environment and contamination of surfaces in close contact to other patients. Indeed, glove misuse is regularly present in all healthcare facilities worldwide, and medical staff often fails to remove gloves between patients or between contacts with various sites on a single patient, thus facilitating the spread of microorganisms [1,2].

Therefore, new tactics to make the general use of medical gloves more feasible and safer without the risk of surface contamination have been increasingly explored during the past years. Indeed, a number of new innovative technologies have emerged to address this issue, such as impregnated glove materials that release chlorine dioxide when activated by light or moisture to produce a disinfecting micro-atmosphere or antimicrobial dye [3,4]. So far, all of such attempts have reached only a clinically insufficient reduction in bacterial counts, even after unrealistically long waiting times and additional physical requirement of light or humidity exposure.

While promising, it remains questionable if an antibacterial examination glove will be able to achieve the ultimate aim of such technologies, which is elimination of cross-infection through contamination of surfaces and patients via contaminated examination gloves [5]. For this, the antibacterial efficacy on the surface of the glove itself would be only successful, if microorganisms contaminating examination gloves are to be killed off or inactivated immediately, hence, within a contact time of 3–5 seconds. This, however, will be impossible to achieve by use of antibacterial coated glove surfaces, as no applicable antimicrobial compound, regardless of its concentration, will achieve a 3–5 seconds claim, leaving aside methodological problems to demonstrate this even under controlled settings such as in a laboratory. As the efficacy of all antimicrobial compounds depend on concentration and exposure time, a method may be applied where an antimicrobial compound will be able to continue its antimicrobial action against transferred microorganisms on the surface touched by the contaminated glove.

Recently, a non-sterile powder free nitrile-based medical examination glove has been developed. This glove has a coating with the active ingredient polyhexamethylene-biguanide hydrochloride (PHMB) on its outside surface. The glove is intended to be worn by medical staff during patient examination and patient care to prevent cross-contamination of microorganisms between clinically relevant contaminated surfaces found in healthcare settings, patients, and other individuals. The present in-vitro experimental study was designed to evaluate the number of bacteria recovered from an initially sterile steel surface after contamination with bacteria originating from a pigskin substrate through coated examination gloves, as compared to identical non-coated control gloves.

## Methods

### Test material and preparation

Irradiated pigskins were used to simulate contaminated skin. Single-use pigskins cut to a size of 40 mm diameter were sterilized by irradiation at a radiation dose at 25 to 35 kGy. The irradiated sterile samples remained frozen at -20°C until final evaluation. One day prior to testing, the pigskins were aseptically placed into a refrigerator at +6°C to thaw. On test day, the sterile and thawed pigskin samples were aseptically placed into separate sterile Petri plates with lids replaced. One day prior to testing, 4 mm diameter coupons made of 304 stainless steel were washed and rinsed with deionized water, allowed to dry, and then sterilized by autoclaving at 121°C. On test day, the sterile stainless steel coupons were placed into separate sterile Petri plates with lids replaced.

Pre-cut swatches of coated and non-coated examination gloves were used as transfer vector for the experiments. Non-sterile powder-free examination gloves were made from synthetic carboxylated acrylonitrile butadiene rubber, coated on their external side with PHMB. Identical but uncoated examination gloves were used as control. All gloves were naturally aged (manufacturing date: July 2011) and provided by Ansell Healthcare Products LLC, Shah Alam, Malaysia.

One day prior to testing, 100 mL of TSB (Tryptic Soy) were inoculated with lyophilized challenge species and incubated at 36°C ± 1°C. On test day, 50 mL of the overnight broth culture was transferred into a centrifuge tube and centrifuged at 5,000 rpm for 5 minutes. The supernatant was discarded and the bacterial pellet was re-suspended in 50 mL of sterile 0.9% Sodium Chloride irrigation, and centrifuged a second time at 5,000 rpm for 5 minutes. Suspensions containing 1 × 10^9^ colony-forming units (cfu) of 4 clinically relevant bacterial species representing Gram-positive (*Enterococcus faecium* ATCC #51559; *Staphylococcus aureus* ATCC #33591) and Gram-negative (*Escherichia coli* ATCC #25922; *Klebsiella pneumoniae* ATCC #4352) bacteria together with 5% BSA serving as organic challenge soil were used to contaminate pigskins. The purity of each challenge suspension was verified on test day by preparing isolation streaks on TSA, incubated at 36°C ± 1°C for 48 hours. The population of each challenge suspension was determined by preparing 10-fold serial dilutions in duplicate.

To demonstrate that the neutralizing solution (Butterfield’s Phosphate Buffer solution with surfactants, BBP++) and media used in testing and plating were capable of adequately neutralizing the antimicrobial properties of the antibacterial treated glove material, neutralization verification was performed using all four challenge bacterial species. Both BBP++ and TSA+ were demonstrated to be effective in neutralizing the bactericidal properties of the antibacterial treated glove versus each species and to be non-toxic to each species.

### Experimental procedure

After contamination of pigskin pieces with an aliquot of 0.05 mL of challenge suspension, swatches of glove material excised from coated and non-coated (control) gloves were firmly pressed onto the inoculated pigskins. The inoculum was allowed to spread evenly across the surface between the pigskin and the test glove swatch by capillary action. A sterile 75 g weight was immediately placed onto the test glove and allowed to remain in place for 1 minute. Following the 1 minute exposure time, the weight was removed and test glove swatch was placed into a sterile Petri plate with the exposed site facing up, remaining undisturbed at ambient temperature for 5 minutes. Then, the test glove swatch was placed onto a sterile 40 mm diameter stainless steel coupon with the contaminated side facing on the stainless steel coupon. A sterile 75 g weight was immediately placed onto the test glove and allowed in place for 1 minute.

The contaminated pigskin, the stainless steel coupon, and the test glove swatch were separately transferred into sterile specimen cups containing 50 mL BBP++, and vortexed thoroughly. The experimental procedure was repeated for coated and non-coated glove swatches and for all 4 test organisms separately in 5 replicates. The type of gloves was not blinded to investigators,

### Sampling processing and data collection

For the pigskin, the stainless steel coupons, and the test glove swatches, 10-fold dilution of each sample was prepared in BBP++, mixed thoroughly using a vortex mixer between dilutions. A 0.1 mL aliquot of the appropriate dilution was pour-plated in duplicate using TSA+, producing plate dilutions of 10^-2^, 10^-3^, 10^-4^, 10^-5^, and 10^-6^. The plates were incubated at 36°C ± 1°C for 48 hours.

Colonies on the plates were counted manually using a hand-tally counter. For counting the number of CFUs, plate dilution with counts in the range of 30 to 300 CFU were used in the data calculation.

### Data calculation and statistical methods

The log_10_ average population and the cfu/mL of the average of the duplicate counts was calculated as log_10_ (C_i_ × 10^-D^) or (C_i_ × 10^-D^), respectively, where C_i_ = average of two plate counts and D = dilution factor of the plates counted.

The population of each challenge species recovered from the surfaces after transfer procedure (P_EX_) was calculated as (1), the log_10_ P_EX_ was calculated as (2), where C_i_ = individual plate count, n = number of plates counted as each dilution, D = dilution factor of counts used, and V_mL_ = volume of diluent at the 10° dilution (in the conducted experiments: 50 mL).

(1) $$P_{\mathrm{EX}}\left(\mathrm{cfu}/\text{surface}\right)=\left(\left(\sum_{\mathrm{Ci}}/n\right)\;x\;10^{-D}{x\;V}_{\mathrm{mL}}\right),$$

(2) $${\mathrm{Log}}_{10}P_{\mathrm{EX}}=\log_{10}\left(\left(\sum_{\mathrm{Ci}}/n\right)\;x\;10^{-D}{x\;V}_{\mathrm{mL}}\right),$$

The log_10_ average P_EX_ (3) recovered from the surface after transfer procedure was calculated as:

(3) $$\log_{10}{\text{average}\;P}_{\mathrm{EX}}=\left(\sum \;\left(\log_{10}P_{\mathrm{EX}}\right)\right)/N,$$

Where N = number of replicates (in the conducted experiments: N = 5) P_EX_ = cfu/surface

Finally, the log_10_ reduction factor (log_10_ RF) for each challenging species was calculated by determining the difference between the log_10_ average P_EX_ recovered from the stainless steel coupons following non-coated control glove transfer and the log_10_ average P_EX_ recovered from the stainless steel coupons following coated glove transfer. Hence, a difference in the bacterial recovery was defined as the mean number of bacteria recovered from the stainless steel coupon by the coated glove, as compared with the mean number recovered from the stainless steel coupon by the non-coated control glove. For viable cfu counts, means were calculated and compared through use of a two-tailed t-test. A p-value of less than 0.05 was considered to be statistically significant.

## Results and discussion

**Figure 1:**
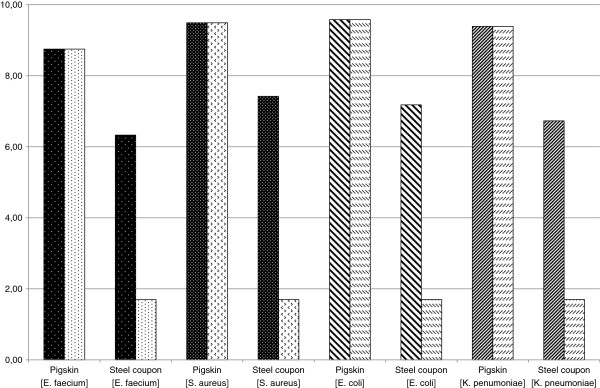
**Pre- and post-exposure populations of challenge microorganisms following transfer procedures.** Left bars: uncoated glove, right bars: coated glove.

The detailed bacterial counts expressed as mean log_10_ cfu, stratified by type of glove and sampled surfaces are summarized in Table 1 and depicted in Figure 1. Regardless of test bacteria, non-coated examination gloves allowed recovery of a mean of log_10_ 6.91 cfu per stainless steel coupon surface after 1 minute direct contact with contaminated skin followed by 5 minutes contamination free rest, and still showed a remaining mean contamination of log_10_ 6.90 cfu per glove surface after 1 minute contact with a sterile stainless steel coupon.

**Table 1:** **Mean log**_**10 **_**cfu/surface of pre- and post-exposure populations of challenge microorganisms following the transfer procedure for antibacterial versus non-antibacterial examination gloves**

	**Initial population, start**		**Pigskin, after contact**			**Coupon, after contact**			**Glove, after contact**		
	**A**	**B**	**A**	**B**	**diff.**	**A**	**B**	**diff.**	**A**	**B**	**diff.**
*E. faecium*	8.75	8.75	7.24	5.55	1.69	6.33	< 1.70	> 4.63	6.30	2.19	4.11
*S. aureus*	9.49	9.49	8.24	6.39	1.85	7.42	< 1.70	> 5.72	7.38	3.00	4.38
*E. coli*	9.58	9.58	7.93	6.57	1.36	7.18	< 1.70	> 5.48	7.05	<1.70	>5.35
*K. pneumoniae*	9.39	9.39	8.01	6.73	1.28	6.73	< 1.70	> 5.03	6.86	2.37	4.49

A = non-treated control glove; B = antibacterial treated test glove.

Results of five replicate experiments, each.

Differently to conventional non-coated examination gloves, coated gloves allowed recovery of a mean of < log_10_ 1.70 cfu per contact surface after 1 minute direct contact with contaminated skin followed by 5 minutes contamination free rest, and showed a remaining mean contamination of log_10_ 2.31 cfu per glove surface after 1 minute contact with a sterile stainless steel coupon. The difference between test and control gloves was highly statistically significant for both, surface contamination and remaining glove contamination (P < 0.001, two-sided T-test).

### Gram-positive test organisms

After contamination of pigskin with a mean initial population of 5.70 × 10^8^ cfu *E. faecium* or 3.15 × 10^9^ cfu *S. aureus*, respectively, a mean of 6.33 log_10_ cfu *E. faecium* were recovered from sterile stainless steel coupons after touching with non-coated examination gloves (control), and < 1.70 log_10_ cfu *E. faecium* after touching with coated examination gloves (log_10_ RF > 4.63).

For *S. aureus*, a mean of 7.42 log_10_ cfu were recovered from sterile stainless steel coupons after touching with non-coated examination gloves (control), and < 1.70 log_10_ cfu after touching with coated examination gloves (log_10_ RF > 5.72).

After a contact time of 1 minute with contaminated skin and 5 minutes rest without any contact, followed by 1 minute contact with a sterile stainless steel coupon, non-coated examination gloves showed a remaining mean contamination of 6.30 log_10_ cfu *E. faecium* or 7.38 cfu *S. aureus* per surface, respectively, while coated examination gloves showed significantly lower contamination with each test organism (*E. faecium*: 2.19 log_10_ cfu per surface; *S. aureus*: 3.00 log_10_ cfu per surface). The difference between contamination of non-coated control and coated treated test glove was highly statistical significant (P < 0.001; two-sided T-test).

### Gram-negative test organisms

Pigskin was contaminated with a mean initial population of 3.85 × 10^9^ cfu *E. coli* or 2.46 × 10^9^ cfu *K. pneumoniae*, respectively. Non-coated examination gloves (control) transferred a mean of 7.18 log_10_ cfu *E. coli* to sterile stainless steel coupons, coated examination gloves < 1.70 log_10_ cfu *E. coli* (log_10_ RF > 5.48).

For *K. pneumoniae*, a mean of 6.73 log_10_ cfu was recovered from sterile stainless steel coupons after contamination by non-coated examination gloves (control), and < 1.70 log_10_ cfu by coated examination gloves (log_10_ RF > 5.03).

After a contact time of 1 minute with contaminated skin and 5 minutes rest without any contact, followed by 1 minute contact with a sterile stainless steel coupon, non-coated examination gloves showed a remaining mean contamination of 7.05 log_10_ cfu *E. coli* or 6.68 cfu *K. pneumoniae* per surface, respectively, while coated examination gloves showed significantly lower contamination with each test organism (*E. coli*: <1.70 log_10_ cfu per surface; *K. pneumoniae*: 2.37 log_10_ cfu per surface). As for Gram-positive organisms, the difference between contamination of non-coated control and coated test gloves with Gram-negative bacteria was also highly statistical significant (P < 0.001; two-sided T-test).

The results of this experimental in-vitro study demonstrated that number of bacteria recovered from initially sterile stainless steel coupons after contact with gloves contaminated on pigskin substrates with high bacterial loads was significantly lower after contact with coated examination gloves, as compared to identical non-coated control gloves. Furthermore, detailed analysis of results revealed that this outcome was not solely due to the antibacterial activity on the surface of test gloves, but may have also be assisted by remaining residues of the coated antiseptic on the source and target surfaces. This observation is supported by the significantly lower recoverable numbers of test organisms on contaminated pigskin substrates after touching them with a coated examination gloves (mean log_10_ cfu reduction = 2.99), as compared to a non-coated identical conventional examination glove (mean log_10_ cfu reduction = 1.44; P < 0.001). The use of an effective neutralizer against PHMB ascertained that the lower cfu counts retrieved from the contaminated pigskin substrates after 1 minute contact with coated examination gloves was not the effect of antiseptic residues being active over prolonged time on pigskin, but that the antiseptic compound PHMB showed an additional reduction in the magnitude of 1.55 log_10_ within 1 minute exposure time on the source surface.

This observation is important as it demonstrates that the coated antiseptic leaches off from the treated antibacterial examination glove. As the efficacy of all antimicrobial compounds depends on concentration and exposure time, the transferred antimicrobial compound seems to be able to continue its antibacterial action against transferred microorganisms on the surface touched by the examination glove. Furthermore, in clinical practice the presence of a neutralizing agent on surfaces may not be assumed, and may even increase the antibacterial efficacy of a coated examination glove.

For an antibacterial examination glove to be successful in decreasing the number of transferred bacteria from one surface to another in a situation simulated by the present experiments, at least four requirements are needed to be fulfilled: first, the used antibacterial compound must be leaching off the glove surface; hence, an antibacterial relevant concentration must be transferred from the glove surface to a touched surface; second, the antibacterial compound must be able to demonstrate an antibacterial efficacy on the surface within a short contact time in order to prevent further contamination of the next glove or healthcare workers’ hand; third, the antibacterial efficacy must not be significantly decreased in the presence of soil or organic matter or presence of neutralizers; and fourth, the used antibacterial compound must not be toxic to humans or interfere with the material and integrity of commonly used surface materials in healthcare settings (lack of corrosion, colour change).

The in-vitro antibacterial action of PHMB is well investigated and published in detail elsewhere [6]. PHMB is a membrane-active, cationic agent whose antibacterial mode of action is based on disruption of the bacterial cytoplasmic membrane and leakage of macromolecular components [6-8]. PHMB binds irreversibly to the surface of bacterial cell membranes and induces a reorganization of the membrane. This mode of action together with the molecules high tenacity resulting in prolonged exposure periods makes it unlikely that microorganisms can develop resistance. Indeed, so far neither induction of resistance nor cross-resistance was demonstrated. An organism either is susceptible to PHMB’s antimicrobial action, or it is naturally unaffected by PHMB (“insusceptible”). Insusceptibility has been noted to some antiseptics (biocides), including PHMB and bacteria of the genus Pseudomonas or Acinetobacter, and is based on bacterial physiology (bacterial cell walls, membrane proteins and efflux pumps, cytoplasmic organelles and cell respiratory processes, enzymes and nucleic acid).

However, because of its cationic nature, one limitation of our experimental work is that the process of coating with PHMB itself may have had an impact by changing the physical surface of the glove and thereby possibly leading to an altered adhesion of microorganisms to the surface. Indeed, recently Moore G et al. [9] identified glove material and glove hydrophobicity as the two most important factors influencing bacterial transfer, with bacterial transfers from gloves to surfaces ranging from 0.01% to 19.5%. If gloves coated with PHMB may have caused such physical effects, our results would still be valid; however, it would be difficult to state that the observed effects were solely the result of an antibacterial mode of action. Secondly, since our experiments were conducted using moist conditions, we are not able to state that such concept of antimicrobial gloves may also demonstrate antimicrobial activity at dry conditions. However, if such glove had not been able to show any antimicrobial activity at moist conditions, then such concept would have to been regarded as irrelevant.

## Conclusion

An experimental in-vitro testing designed to mimic cross-contamination of selected clinically relevant Gram-positive and Gram-negative bacteria in a simulated healthcare setting following glove contact with a contaminated biological surface and cross-transfer to a stainless steel surface has demonstrated that a PHMB coated antibacterial examination glove is able to reduce cross-contamination by > 4 log_10_, compared to a control non-coated examination glove. Based on these results, the use of antibacterial examination gloves may be a justifiable measure to prevent or reduce cross-contamination and indirect transmission of pathogens in the healthcare setting. These benefits may outweigh theoretical risks such as promotion of false use of examination gloves by incorrect assumptions of an extra protection, induction of allergies, or selection of naturally insusceptible bacteria, such as *Pseudomonas spp.* or *Acinetobacter spp*. Based on a well balanced risk-benefit assessment, the findings encourage further clinical investigation on the clinical impact of an antibacterial examination glove in practice.

## Abbreviations

ATCC: American type culture collection; BBP++: Butterfield’s Phosphate Buffer solution with surfactants; BSA: Bovine serum albumin; CFU: Colony forming unit; g: Gram; kGy: Kilogray; mL: Milliliter; mm: Millimeter; PHMB: Polyhexamethylene-biguanide hydrochloride; RPM: Rotation per minute; TSB: Tryptic soy broth; TSA+: Tryptic soy agar with product neutralizer; WHO: World Health Organization.

## Competing interests

JL, RS, VD, and OA have no personal or financial relationship or competing interests to declare. AHE, BNY, and YPT are employed by Ansell Healthcare Products LLC. Some material contained in this manuscript details certain intellectual property rights which are owned by Ansell, Inc. The data presented in this study have been analyzed and compiled by the authors. The conclusions made do not necessarily represent the views or opinions of Ansell Healthcare Products LLC.

## Authors’ contributions

OA, AHE, and BNY have planned and designed the experimental procedures of this study. The study was coordinated and analyzed with support from all authors. AHE, BNY, and YPT managed and collected the data. JL, RS, VD, and OA performed the interpretation and statistical analysis of data and drafted the manuscript. All authors read and approved the final manuscript.
